# An easy and versatile 2-step protocol for targeted modification and subcloning of DNA from bacterial artificial chromosomes using non-commercial plasmids

**DOI:** 10.1186/1756-0500-5-156

**Published:** 2012-03-20

**Authors:** Heiner Hartwich, Hans Gerd Nothwang

**Affiliations:** 1 Department of Neurogenetics, Institute of Biology and Environmental Sciences, Carl von Ossietzky University, Carl-von-Ossietzky-Straße 9-11, 26129 Oldenburg, Germany; 2 Research Center Neurosensory Science, Carl von Ossietzky University Oldenburg, Carl-von-Ossietzky-Straße 9-11, 26129 Oldenburg, Germany

## Abstract

**Background:**

Promoter-specific expression of foreign DNA in transgenic organisms often relies on bacterial artificial chromosomes (BACs). This approach requires modification and subcloning of BAC-DNA by recombineering technologies in *Escherichia coli*. Most current protocols rely on commercial kits or isolation of BACs, their transfer between different host strains, and their restriction.

**Findings:**

In this report we present a 2-step protocol for efficient modification and subcloning of DNA from bacterial artificial chromosomes using the non-commercial plasmids pKM208 and pTP223, distributed from addgene.com. A targeting cassette was successfully integrated into a BAC and 42 kb of this construct were subcloned. Both a plasmid-derived substrate with longer homology arms and a PCR-generated substrate with short homology arms (50 bp) were used for recombination. pKM208 and pTP223 contain all required genes for recombineering, but differ in their antibiotic resistance genes. This makes the system independent of the selection markers on the DNA molecules targeted for recombination.

**Conclusions:**

The time and cost saving protocol presented here compares favorably to currently used systems. Using non-commercial plasmids, it allows targeted modification and cloning of large DNA (> 40 kb) fragments *in vivo *without restriction and ligation. Furthermore, both steps are performed in the same host eliminating the need to isolate BAC DNA and to use different bacterial strains.

## Background

Bacterial artificial chromosomes (BACs) are low copy plasmids based on the F-plasmid of *E. coli*. They can carry up to 300 kb of foreign DNA. The large insert size has made BACs an important source for the generation of transgenic organisms, because they carry entire genes including their regulatory elements. This can be used to express foreign genes in a spatiotemporal pattern, which mimics endogenous gene expression [1-4]. BACs are propagated in recombination deficient *E. coli *strains to achieve high clonal stability and low rate of chimerism.

Methods to introduce genetic modifications into BACs such as insertions, deletions, or point mutations are primarily based on homologous recombination between the BAC and targeting molecules and do not require restriction endonucleases or DNA ligases. The most popular approach for the *in vivo *manipulation of DNA molecules is called recombinogenic engineering or simply recombineering [5,6]. Two main recombineering systems are currently employed. One system is based on the proteins RecE and RecT from the prophage Rac [7]. The second system makes use of Redα, Redβ, and Redγ, encoded by the λ phage Red operon [8]. The exonucleases RecE and Redα generate ssDNA overhangs, to which the DNA annealing proteins RecT and Redβ bind. The interaction between RecE and RecT or their functional analogues Redα and Redβ is required to initiate recombination between short homologous regions. Finally, Redγ prevents the RecBCD-dependent degradation of the targeting DNA molecule [9].

Recombineering systems have been used to engineer the chromosome of *E. coli *[7,8,10-12], the chromosome of other bacterial pathogens such as *Serratia, Shigella, Yersenia, Salmonella *and *Pseudomonas *[13-17], and to manipulate BACs [18,19]. For BAC manipulation, two major systems exist. One is the commercially available Red/ET^® ^system from Gene bridges. The second one consists of a publically available system developed by Copeland and coworkers [19], and requires isolation and transformation of BACs into different host strains.

**Figure 1:**
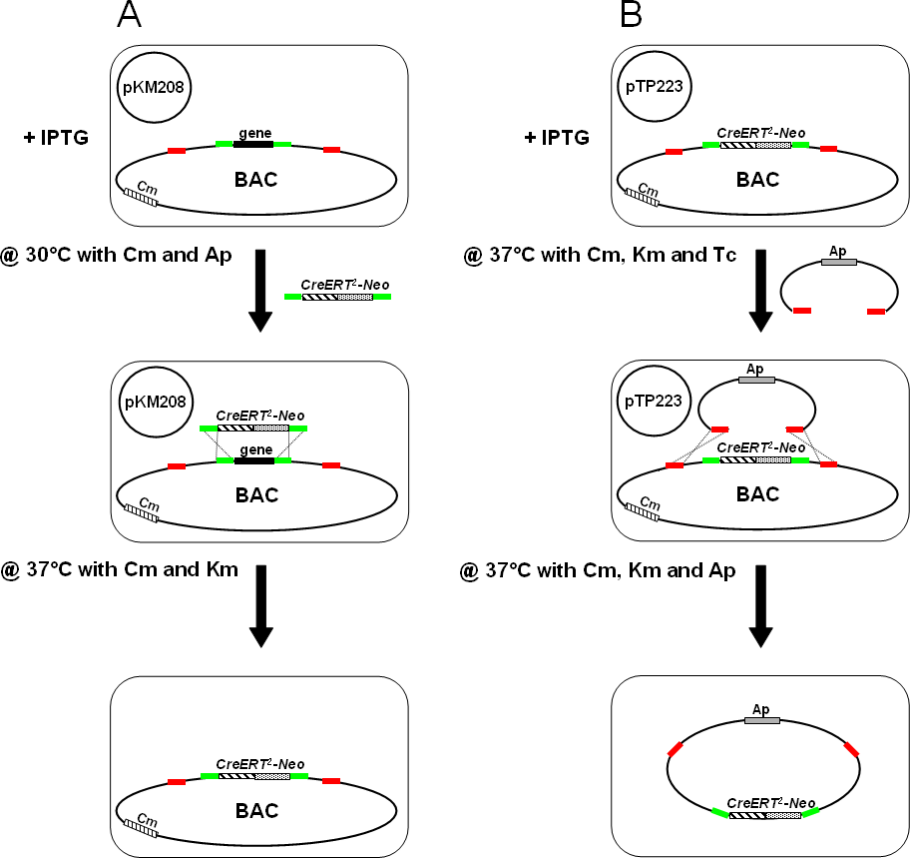
**Schematic representation of the 2-step protocol of recombineering**. (A) After induction with IPTG, BAC and pKM208 containing cells were electroporated with a *CreERT^2^-Neo *cassette with flanking homology arms. This resulted in substitution of the open reading frame by the targeting cassette using homologous recombination. (B) Cells hosting recombined BAC and pTP223 were electroporated with a pBR322-derived targeting cassette. This resulted in subcloning of a large BAC-derived DNA fragment into pBR322. Homologous regions are colored. Note, that DNA fragments are not drawn to scale.

Here we employ successfully the non-commercial plasmids pKM208 and pTP223 (available from addgene.com) in an easy 2-step recombineering protocol. Both plasmids contain the open reading frames for Redα, Redβ and Redγ, but differ in the origins of replication and the selection markers. The low copy plasmid pKM208 has the temperature-sensitive origin pSC101 and confers ampicillin (Ap) resistance, whereas the high copy plasmid pTP223 confers resistance to tetracycline (Tc) [12]. Both plasmids express constitutively the LacI repressor for isopropyl β-D-1-thiogalactopyranoside (IPTG) inducible expression of the recombination enzymes [12]. This feature is useful to estimate the false positive rate by analyzing non-induced cells, and to shut down the recombination system after successful recombineering to minimize undesired rearrangements. We exemplified the successful use of these plasmids by replacing the open reading frame (ORF) of *Math5 *(murine atonal homologue 5) in the BAC RP23-328P3 by a *CreERT^2^-Neo *cassette and subsequent subcloning of the novel 42 kb transcription unit by gap repair (Figure 1).

## Materials and methods

### Transformation of BAC hosts with Red-expressing plasmids

A 20 ml culture from a single BAC carrying host colony was grown to an OD_600 nm _of 0.5 in the presence of chloramphenicol (Cm) (12.5 μg/ml) on a shaker at 37°C. After centrifugation at 6,000 rpm for 10 min, cells were resuspended in 20 ml precooled deionized water. After another round of centrifugation, cells were resuspended in 50 μl precooled deionized water and electroporated with 100 ng of pKM208 ([12]; addgene.com, plasmid 13077) or pTP223 ([20]; addgene.com, plasmid 13263) using a Gene Pulser (Bio-Rad Laboratories, Munich, Germany). Thereafter, pKM208 electroporated cells were incubated in 1 ml LB broth for 1 h at 30°C to account for the temperature-sensitive origin of replication, whereas pTP223 containing cells were incubated at 37°C. After plating, the selective LB-agar plates were incubated at the appropriate temperatures (30°C or 37°C).

### Preparation of electrocompetent cells for recombination experiments

BAC hosts carrying pKM208 were grown at 30°C in 50 ml LB broth, containing Cm (12.5 μg/ml) and Ap (100 μg/ml), whereas BAC hosts carrying pTP223 were grown at 37°C in the presence of Cm (12.5 μg/ml), kanamycin (Km) (25 μg/ml) and Tc (10 μg/ml). After they reached an OD_600 nm _of 0.2 - 0.3, cultures were split into two 25 ml cultures. One culture was supplemented with IPTG to a final concentration of 1 mM, whereas the other culture served as a negative control. After 1 h of shaking, cells containing pKM208 were heat-shocked for 15 min at 42°C and kept on ice to stimulate subsequent gene replacement [12]. This step was omitted for cultures containing pTP223. After centrifugation of 20 ml culture at 6,000 rpm for 10 min, cells were resuspended in 20 ml precooled deionized water. This step was repeated once. Finally, cells were resuspended in 50 μl precooled deionized water and kept on ice until use.

### Generation and of the plasmid-derived targeting cassette for modifying the BAC

The plasmid pMath5-CreERT^2^-Neo was constructed by cloning a PCR product containing the *CreERT^2^-Neo *from pl451-CreERT^2 ^into pMECA [21]. This *CreERT^2^-Neo *cassette was flanked by two homology arms derived from the *Math5 *gene of the BAC RP23-328P3. After validation by sequencing, a 5,107 bp long fragment, containing *CreERT^2^-Neo *and flanking 708 bp and 372 bp long homology arms, was released by restriction with *Dra*I (MBI Fermentas, St. Leon-Rot, Germany) and gel-purified using peqGOLD Gel Extraction Kit (Peqlab, Erlangen, Germany).

### PCR based generation of a pBR322-derived targeting cassette for subcloning

To generate the targeting cassette for subcloning, a PCR was performed with Phusion-Polymerase (New England Biolabs, Frankfurt am Main, Germany) using pBR322 as template and the primers pBR322-Math5-R 5'-*TGTCTCCCACAGTACCTAACATAGGATCTTACAGAGTAGACACACATGAT*GTTTAAACGATACGCGAGCGAACGTG-3' and pBR322-Math5-L 5'-*CCTGCAGCACAGCCTCCAACAGTTTTCAGTCTACTCATCTTTCCTAGTAT*GTTTAAACTTAGACGTCAGGTGGCAC-3' (an *Mss*I site is underlined, *Math5 *homology regions are in italics). After *Dpn*I (MBI Fermentas) restriction, the PCR product was gel-purified using peqGOLD Gel Extraction Kit (Peqlab) and eluted with deionized water.

### Electroporation of targeting constructs

The gene targeting DNA (300 ng) was mixed with 50 μl of cell suspension and put into a precooled 0.1-cm electroporation cuvette and electroporated with a Gene Pulser (Bio-Rad) at 1.8 kV, 200 Ohm, 25 μF for ~ 4.5 ms. Cells were resuspended in 1 ml LB broth, incubated for 1 h at 37°C on a shaker, and then plated on selective LB-agar plates.

### Screening for recombinant clones

Recombinant clones were screened by a junction PCR for probing the insertion of the *CreERT^2^-Neo *cassette, using the targeting cassette specific primer 5'-CTCCTGTCATCTCACCTTGC-3' and the BAC specific primer Math5_90718rev 5'-CCGGACGCATTCAACATCAC-3' (expected product size: 1.6 kb). For further validation, additional junction PCRs were performed. Primers were as follows: Math5_90718rev and 5'-GGCCCGCGCTGGAGTTTC-3' (3.9 kb), Math5_90718rev and 5'-CCACTGCGGGCTCTACTTC-3' (2.9 kb), as well as with the primers 5'-CCTGACACCCTTTTAGTTAAG-3'and 5'- CCATGAGTGAACGAACCTGGTCG-3' (2.6 kb). For gap repair mediated subcloning, DNA from positive clones was purified by alkaline lysis and analyzed by restriction with *Mss*I (MBI Fermentas).

## Results and discussion

To establish an easy, cheap, and fast protocol for recombination-based modification of BACs, we assessed the non-commercial plasmids pKM208 and pTP223. Both plasmids have been successfully used for engineering of bacterial chromosomes [10,12,16], but have not yet been employed for the manipulation of BACs. We tested both plasmids, as their different resistance genes provide flexibility with respect to the selection markers on the targeting constructs and substrates.

### Replacement of the Math5 ORF using pKM208

**Figure 2:**
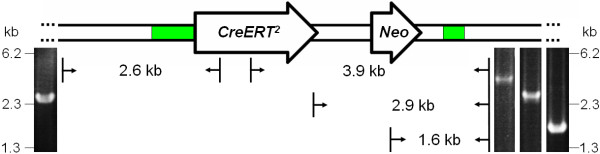
**Analysis of the modified BAC by junction PCRs**. Schematic representation of the performed junction PCRs is depicted. The *CreERT^2^-Neo *targeted region of the BAC with the corresponding primer binding sites (arrows) and the expected sizes of the PCR products are indicated. Homologous regions are depicted in green. Agarose gel electrophoresis demonstrated that all four junction PCRs yielded products of the correct size.

To probe the potential of the plasmids to modify BACs, DH10B cells containing pKM208 and RP23-328P3 were transformed with a 4 kb *CreERT^2^-Neo *cassette with flanking homology arms of 708 bp and 372 bp (Figure 1A). Only IPTG induced cells gave rise to colonies on double selecting plates (Cm and Km), confirming previous data on the tightly controlled gene expression from this vector [12]. Correct homologous recombination was verified by a junction PCR (Figure 2). 13 out of 50 colonies (26%) were positive (data not shown). One positive clone was further validated by three additional junction PCRs (Figure 2) and sequencing of the resulting products. To prevent undesired subsequent recombination events in the modified BAC, the temperature-sensitivity of the low-copy origin pSC101 was exploited. After heat induction, loss of pKM208 was demonstrated by the lack of Ap resistance (data not shown). These results demonstrate that pKM208 is well suited for homologous recombination using a BAC template and a plasmid-derived substrate.

### Subcloning of DNA from the BAC using pTP223

It is often advisable to use only part of a BAC to generate transgenic organisms. This will prevent expression of additional genes residing in the BAC, which would result in increased gene dose, often entailing abnormal phenotypes [22,23]. This can be achieved by two different approaches. The desired transcriptional unit can be released using appropriate restriction enzymes. Alternatively, it can be subcloned by gap repair using pBR322 [19], avoiding tedious gel-purification. We therefore tested next, whether the plasmids could mediate gap repair-based subcloning. For this purpose, primers were designed with a 50 nucleotide long sequence homologous to the target sequence in the BAC, an *Mss*I site, and 20 nucleotides, that match to pBR322 [19]. The *Mss*I sites were added for easy release of the entire insert for transgenic approaches. This time we tested pTP223 (Figure 1B).

**Figure 3:**
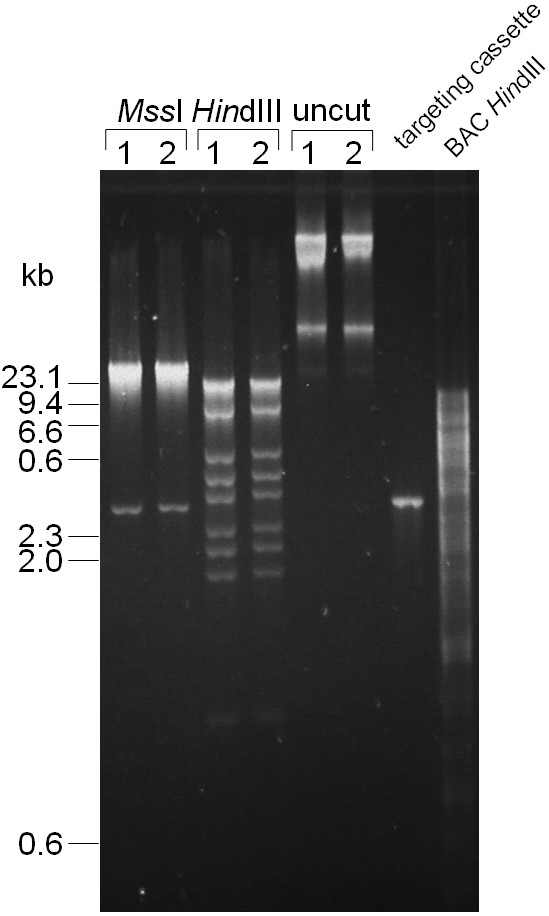
**Analysis of the final construct**. Two *CreERT^2^-Neo *positive clones were analyzed by restriction with *Mss*I (lanes 1 and 2). This resulted in the 42 kb insert (upper band) and the 2.8 kb long backbone (lower band). The restriction of the two constructs with *Hin*dIII resulted in the expected restriction pattern of 17.9 kb 7.9 kb, 4.5 kb, 3.6 kb, 3.1 kb, 2.5 kb, 2.2 kb, 1.9 kb and 1 kb (lanes 3 and 4). Uncut clones are shown in lanes 5 and 6. In lane 7, the PCR generated targeting cassette of 2.9 kb is shown, which has almost the same size as the 2.8 kb released backbone after restriction with *Mss*I. The restriction of the *CreERT^2^-Neo *targeted BAC RP23-328P3 with *Hin*dIII resulted in fragments ranging from 13 bp to 14.9 kb (lane 8). The correct restriction pattern for both *Mss*I and *Hin*dIII demonstrated successful targeting and subcloning of the final construct.

DH10B cells containing the modified BAC and pTP223 were transformed with the PCR-generated pBR322-derived targeting cassette to subclone a 42 kb fragment containing the modified transcription unit (*Math5::CreERT^2^*). 24 colonies from induced cells were analyzed by a plasmid preparation followed by restriction with *Mss*I. This resulted in 2 putative positive clones, which were confirmed by restriction using *Hin*dIII (Figure 3). The low efficiency of homologous recombination compared to our replacement experiment and a previous report [24] is likely due to the large size of the heterologous region and the short homology arms used in this experiment. Taken together, these data demonstrate that pTP223 promotes recombination with short homology arms (50 bp in length), which is in agreement with recent data [9]. In the absence of IPTG, no transformants were observed on Ap and Km containing LB agar plates, demonstrating tight regulation of the Red operon by IPTG in this plasmid as well.

Since spontaneous loss of pTP223 has been reported [12], we tested positive clones on Tc containing LB-agar. Absence of growth revealed that pTP223 was lost after recombination. This beneficial feature allows stable propagation of the constructs.

## Conclusions

We report a protocol for the modification and subcloning of large BAC-DNA by the use of the non-commercial plasmids pKM208 and pTP223. The protocol sidesteps restriction and gel-purification of large DNA fragments. Furthermore, no BAC isolation and transformation into other strains like DY380, EL250 and EL350 is required [19]. Both plasmids are available at addgene.com and are an example for the high potential of shared sources to establish and adapt low cost protocols for everybody's use.

Here, we used pKM208 for targeting the BAC with a plasmid-derived substrate and pTP223 for subcloning. This was done to demonstrate the potential of both plasmids to promote homologous recombination using BACs as template. In future studies, only one of the two plasmids is sufficient. The choice of the plasmid solely depends on the selection marker, which should be different from those present in the BAC and the DNA substrate for homologous recombination. The availability of two different selection markers renders the system very flexible and versatile. Finally, selection for these markers in the media will ensure recombineering potential in all cells of the culture.

## Competing interests

The authors declare that they have no competing interests.

## Authors' contributions

HH conceived and carried out the experiments and drafted the manuscript. HGN discussed the results with HH and helped to draft the manuscript. Both authors read and approved the final manuscript.
